# An Innovative Master Haptic Interface Employing Magnetorheological Fluids for Endovascular Catheterization

**DOI:** 10.3390/s25247450

**Published:** 2025-12-07

**Authors:** Linshuai Zhang, Siyu Huang, Jinshan Zuo, Shuoxin Gu, Lin Xu, Yujie Zhang, Tao Jiang

**Affiliations:** 1 School of Intelligent Medicine, Chengdu University of Traditional Chinese Medicine, Chengdu 611137, China; zhanglinshuai@cdutcm.edu.cn (L.Z.); vennrisper@stu.cdutcm.edu.cn (S.H.); zuojinshan794@gmail.com (J.Z.); xulin@cdutcm.edu.cn (L.X.); zhangyujie@cdutcm.edu.cn (Y.Z.); 2 School of Automation, Chengdu University of Information Technology, Chengdu 610225, China; gsx@cuit.edu.cn; 3 Engineering Research Center of Traditional Chinese Medicine Artificial Intelligence of Sichuan Province, Chengdu 611137, China

**Keywords:** master haptic interface, magnetorheological fluids, endovascular catheterization, safe operation, vascular interventional surgery

## Abstract

Inadequate force feedback and collision warnings in teleoperated surgical instruments elevate risks during intravascular cannulation. This study introduces an innovative master haptic interface that utilizes magnetorheological (MR) fluid to enhance surgeons’ operational perception during robot-assisted intervention surgery. The system delivers real-time haptic feedback to enhance surgical operational safety and automatically amplifies the feedback force when the contact force on the slave side surpasses the predefined threshold, enabling timely collision alerts. A series of preliminary experiments has been carried out to validate the efficacy of this particular type of haptic interface. The experimental results clearly indicate that the master haptic interface based on MR fluid and carefully designed can effectively enhance the operator’s haptic perception and provide collision alarms in a timely manner with haptic clues, improving the safety and operability of robot intravascular intervention. This research provides some insights into the functional improvements of safe and reliable robot-assisted catheter systems.

## 1. Introduction

According to a report by the American Heart Association (AHA), cardiovascular and cerebrovascular diseases have become one of the three major causes of human death (heart disease, stroke and vascular diseases). Even in developed countries, the number of deaths from cardiovascular disease is as high as 34% every year [1]. The number of patients with sudden cardiac death due to arrhythmia is increasing annually. If they cannot be treated effectively in time, it will lead to myocardial infarction and stroke, directly leading to death, which is a serious threat to human health.

With the rapid advancement of medical technology, vascular interventional procedures have become a widely adopted approach to diagnose and treat various cardiovascular diseases, including arterial stenosis, thrombosis, and atherosclerosis. Conventional cardiac interventional procedures typically involve percutaneous insertion of flexible catheters and guidewires through the femoral or radial artery, which are navigated along the vascular pathway to the target lesion under the visual guidance of a digital subtraction angiography (DSA) system [2]. However, these procedures present several challenges. Prolonged manual operations can result in surgeon fatigue and involuntary tremors, potentially compromising surgical precision. Moreover, repeated exposure to ionizing radiation increases the risk of occupational hazards, including cancer and cataracts, even with the use of protective equipment [3]. Notably, certain body parts, such as the hands and face, remain inadequately shielded, and the extended use of heavy protective garments often leads to musculoskeletal strain. To address these limitations, the field of interventional cardiology has undergone significant technological evolution. Percutaneous coronary intervention (PCI) has become the preferred revascularization strategy for patients with coronary artery disease (CAD) [4,5]. Despite its clinical efficacy, PCI and other catheter-based procedures expose interventional cardiologists to substantial occupational risks, particularly radiation [6]. Increasing awareness of these hazards has catalyzed the integration of robotic systems in cardiovascular interventions [7,8]. Robotic platforms not only minimize radiation exposure but also offer enhanced motion precision, enabling consistent submillimeter-scale translation and fine rotational control, which are often challenging for human operators [9,10,11,12].

In recent years, robot-assisted technology has become increasingly important in the medical field. To address these challenges, numerous research institutions and commercial entities have integrated robot-assisted technology with vascular interventional surgery, resulting in the development of various robot-assisted operating systems for intravascular procedures. These systems effectively mitigate the issue of cumulative radiation associated with traditional vascular interventional surgery, enhance stability and precision, and reduce surgical risks. Efforts related to robot-assisted vascular interventional surgery (VIS) have primarily concentrated on the design of master–slave assistance [13,14] and the implementation of haptic feedback [15,16]. In the master–slave assistance domain, a substantial number of robotic systems have been proposed to facilitate robot-assisted surgeries. Yang et al. [17] introduced a master–slave interventional surgical robot based on an isomorphic interactive device, capable of executing complex operations in remote surgery. However, their compact design requires a large spatial volume. Bao et al. [18] developed a telerobotic system utilizing two commercial haptic devices (TouchTM X, USA), which was validated through both laboratory and in-human experiments, albeit without considering the surgeon’s inherent surgical skills [19]. Kundrat et al. [20] designed an endovascular robotic platform that provided insights for clinical translation with device deployment using an MR-safe teleoperation platform, although the master device is limited to a single instrument. Wang et al. [21] presented a 12-degree-of-freedom (DOF) insertion system with increased redundancy to facilitate the movement of surgical instruments. Chen et al. [22] proposed a magnetic microactive guidewire for surgical applications to reduce operating time, though it proved challenging to insert into vessels with complex shapes due to size constraints. Overall, these studies predominantly focused on the use of rocker devices (either commercial or self-designed) or single-instrument control, with limited attention to multi-instrument manipulation and the natural operating habits of surgeons within master–slave systems. Consequently, this research effort introduces a novel VIS employing coaxial operation to accommodate natural operation and multi-instrument control.

In traditional intravascular interventional surgery, experienced surgeons rely on tactile feedback from the catheter tissue by sensing the subtle axial force and torque at the fingertips when maneuvering surgical instruments such as catheters and guidewires through various patient arteries. The integration of real-time imaging data aids in mitigating the risk of vascular perforation at the bends by allowing strategic insertion, retraction, and rotation of the instruments at the proximal end. However, in robot-assisted vascular interventional surgery, surgeons are unable to directly manipulate tools and obtain haptic feedback. Visual assistance alone is insufficient to ascertain potential collisions within areas of vascular curvature [23]. Consequently, some researchers have incorporated force measurement devices into robotic catheter systems to provide a more intuitive representation of force interactions with blood vessels during intravascular procedures. As force sensor technology advances towards miniaturization and increased precision, the deployment of force/torque sensing devices is crucial for the remote operation of robotic catheter systems. These devices can alert surgeons to promptly adjust the direction of the surgical tools when the catheter tip contacts the vessel wall. For instance, a linear motor has been utilized to generate haptic feedback [24], facilitating integrated haptic feedback. A highly sensitive, compact visual force sensor [25] was designed to capture force signals, offering more intuitive force feedback. Nevertheless, its effectiveness is constrained by the lack of a comprehensive robot-assisted validation. Zhou et al. [26] designed a master manipulator based on surgeons’ habits, functioning as a single operating unit with both axial and circumferential haptic feedback on the master side, which aids in the tactile implementation of robot-assisted vascular interventional surgery (VIS). However, the operating handle significantly differs from actual medical instruments.

Despite notable progress in vascular interventional robotics, the integration of effective haptic feedback remains a significant challenge, primarily manifesting in two key areas. First, the prevailing paradigm relies on visual force monitoring, where surgeons must continuously interpret sensor data from a screen to infer collisions. This process induces considerable cognitive load and operator fatigue, diverting attention from the primary visual field. Second, existing commercial force feedback systems, often based on fixed, motor-driven actuators, are frequently criticized for insufficient safety, stability, and a lack of natural interaction. They fail to replicate the intuitive feel of manipulating real surgical instruments, creating a barrier to seamless robotic adoption.

In this study, a master haptic interface leveraging magnetorheological (MR) fluid is introduced, whose innovation is benchmarked through superior actuation and ergonomic design. The system fully exploits the unique rheological properties of MR fluids, enabling stepless, stable, and precisely adjustable force feedback through controlled magnetic fields—a significant advantage over conventional motors. In addition, compared to the previous design of our research group [15,27], the rotating cylinder combined with the bearing as a haptic force feedback module can minimize the friction interference from the MR fluid container, thereby ensuring high-fidelity tactile sensation while providing a wider force range. The design of the interface is in line with the surgeon’s operation habits, which can maximize the operation skills obtained by the surgeon after training and provide an immersive and on-site operation feeling. This direct haptic connection not only enhances realism but also proactively aids in identifying when forces exceed vascular safety thresholds, thereby substantially elevating procedural safety compared to systems that rely on indirect visual force cues.

## 2. System Architecture

**Figure 1:**
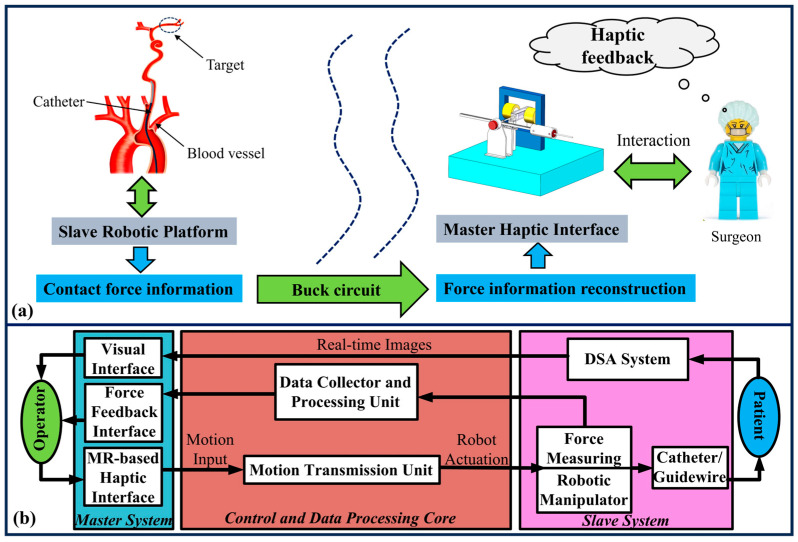
Overview of the endovascular robot-assisted catheter/guidewire operating system: (**a**) Schematic diagram of the proposed robot-assisted catheter/guidewire operating system. (**b**) Signal flow chart of the proposed robot-assisted catheter/guidewire operating system.

Figure 1 illustrates the overall architecture of the master–slave endovascular robot-assisted catheter/guidewire operating system, which is structured as a closed-loop control system. As shown in Figure 1a, the system integrates haptic feedback and interaction through a Master Haptic Interface operated by the surgeon, enabling realistic simulation of conventional surgical maneuvers. The complete system architecture, depicted in Figure 1b, consists of three core components: the Master System, the Slave System, and the Control and Data Processing Core, forming a seamless master–slave control loop.

The master system includes the master haptic interface, a visual interface, and a force feedback interface. The surgeon manipulates the catheter/guidewire through the haptic device, generating motion inputs. The system also provides real-time visual and force feedback to enhance operator awareness and precision. The slave system comprises a robotic manipulator equipped with a force-sensing unit and an imaging navigation system (e.g., DSA System). The slave unit accurately replicates the motion commands from the master side, advancing the catheter/guidewire inside the patient’s blood vessels. It also measures contact forces and collects real-time images. The control and data processing core serves as the central bridge between the master and slave subsystems. It includes a motion transmission unit for sending motion commands, a data collector and processing unit for handling sensor data, and a force information reconstruction module. This core ensures synchronized motion control and feedback of both force and positional data. The operator manipulates the catheter/guidewire via the master haptic interface. Motion signals are transmitted through the motion transmission unit to the slave robotic manipulator, which reproduces the movements inside the patient’s vasculature. Simultaneously, real-time images from the imaging system provide visual guidance. A high-precision force sensor on the slave side captures contact forces between the catheter and vessel walls. This force data is reconstructed and fed back to the master side, displayed both graphically on a PC interface and felt through the haptic device.

## 3. Design of the Novel Master Haptic Interface

**Figure 2:**
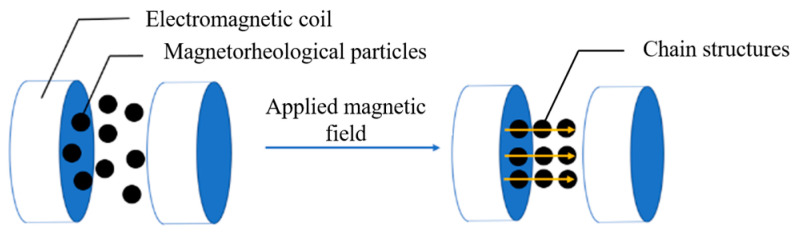
The magnetic field characteristic of magnetorheological particles.

Magnetorheological fluid is a specialized controllable fluid, consisting of magnetic particles characterized by high permeability and low hysteresis when suspended in a nonmagnetic liquid. As illustrated in Figure 2, in the absence of a magnetic field, the magnetic particles within the magnetorheological fluid are randomly and uniformly dispersed throughout the liquid, resulting in a viscous flow that behaves as a Newtonian fluid with low viscosity. Upon application of an external magnetic field, the fluid exhibits a distinct magnetorheological effect, altering its magnetic chain structure. The magnetic particles align to form chains with a relatively stable structure along the direction of the magnetic field lines, and these chains become increasingly solid and stable as the magnetic field strength increases. Under the influence of the magnetic field, the viscosity of the magnetorheological fluid increased significantly, exhibiting solid-like characteristics as the magnetic field strength increased. This transformation is reversible; when the magnetic field is removed, the magnetorheological fluid reverts to its original fluid state.

**Figure 3:**
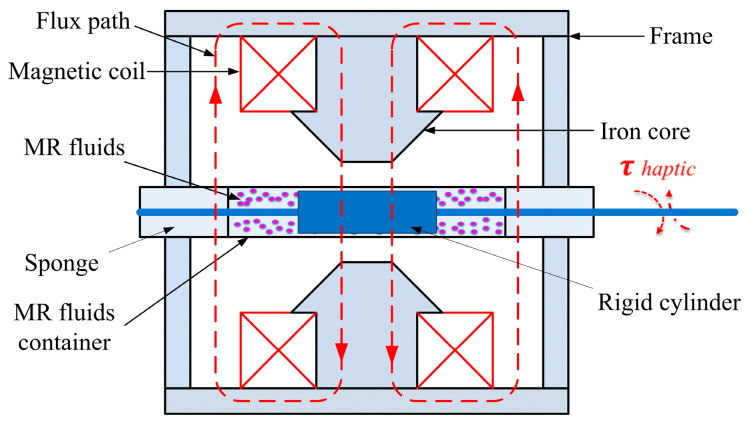
Schematic of the haptic master device.

In conjunction with the properties of magnetorheological (MR) fluids in a magnetic field and the tactile requirements of the robot-assisted catheter system, resistance torque is generated between the MR fluids and a rigid cylinder rotating within the MR fluids under the influence of a magnetic field (see Figure 3). The application of a magnetic field induced a shear resistance moment on the rotating cylinder situated within the MR fluid. The shear resistance moment varies with changes in the magnetic induction intensity applied to the MR fluid. Consequently, the resistance moment output of the actuator can be adjusted by modulating the magnetic induction intensity within the working gap.

**Figure 4:**
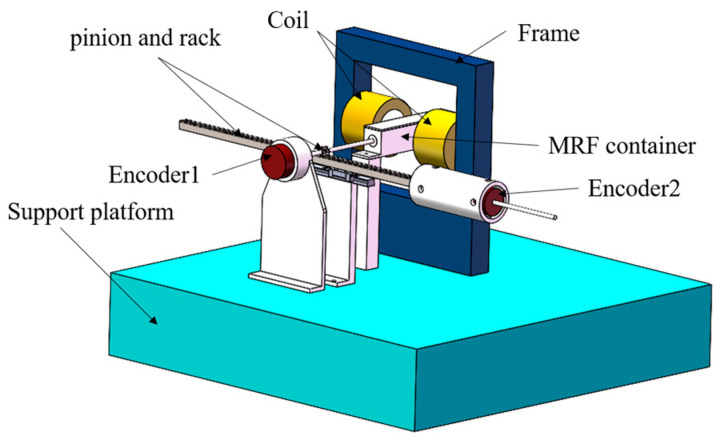
A prototype of the master haptic interface.

Based on an analysis of the characteristics of magnetorheological (MR) fluids and haptic feedback requirements of the robotic catheter operating system, a prototype of the master haptic interface is proposed, as illustrated in Figure 4. This prototype comprised two electromagnetic coils with iron cores and an MR fluid container. The detailed design specifications for the haptic interface are introduced in [28]. The specific concentric coil configuration was chosen to create a uniform and concentrated magnetic field across the MR fluid gap, maximizing the achievable yield stress while minimizing the device’s volume. The magnetic circuit was meticulously optimized using Finite Element Analysis (FEA). The simulations were critical for iterating the core geometry, pole piece shapes, and coil placement to achieve the target magnetic flux density within the MR fluid volume. The advantage of this novel design lies in its ability to mitigate the influence of friction between the operating rod and magnetorheological fluid container on the haptic force. A pinion-and-rack mechanism coupled with two rotary encoders was employed. These encoders were utilized to capture and transmit translational and rotational signals to the slave manipulator during the procedure. When the catheter collides with the vascular wall and the contact force measured at the slave manipulator exceeds the corresponding threshold, the master haptic interface promptly generates resistance as a collision warning to alert the operator, thereby preventing vessel puncture.

## 4. Establishment of Mechanical Model of the Master Haptic Interface

The mechanical structure design of the master haptic interface based on a magnetorheological fluid (MRF) is crucial in determining the device performance indicators, including adjustable accuracy, response speed, real-time performance, and mechanical loss. A well-conceived structural design and modeling of the master haptic interface are essential for analysis and parameter correction, which significantly contribute to the optimization of the structural design and the enhancement of feedback effects. In both domestic and international academic research, the Bingham model was recognized as one of the earliest and most extensively utilized models. Under a steady-state shear field, the stress–strain relationship of the magnetorheological fluid is characterized as follows:

(1) $$\tau =\tau_{H}+\eta \gamma ,\tau \geq \tau_{H}$$

**Figure 5:**
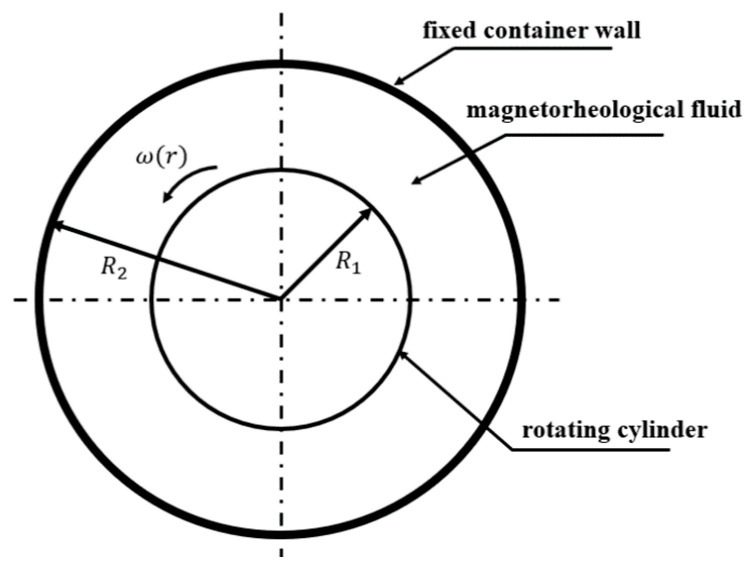
Master haptic operating interface model.

In the formula, τ is the shear stress of the MR fluid; $\tau_{H}$ is the dynamic yield stress of the MR fluid; η is the viscosity coefficient of the MR fluid; and γ is the shear rate. A model for the master haptic manipulator is shown in Figure 5.

In Figure 5, *R*_1_ and *R*_2_ are the radii of the rotating cylinder and the fixed container, and the magnetorheological fluid is filled between them. When the rotating cylinder rotates at an angular velocity ω, the magnetorheological fluid is rotated by shearing with an angular velocity of ω(r).

The resistance torque analysis is carried out as follows:

When an external magnetic field is applied, the fluid transfer torque at a radius r is

(2) $$T=2\pi r^{2}L_{e}\tau_{r}\theta$$

In the formula, $L_{e}$ is the effective length along the axial direction of the magnetorheological fluid under the action of the magnetic field that can produce the magnetorheological fluid effect.

Combined with the relevant structural parameters of the master haptic manipulator designed in the literature, we can obtain:

(3) $$T=2\pi L_{e}C_{1}$$

C_1_ can be represented as:

(4) $$C_{1}=\frac{2\eta R_{1}^{2}R_{2}^{2}}{R_{2}^{2}-R_{1}^{2}}(\frac{\tau_{H}}{\eta }\ln\frac{R_{2}}{R_{1}}+\omega )$$

Substituting Equation (4) into Equation (3), we can obtain:

(5) $$T_{1}=\frac{4\pi L_{e}R_{1}^{2}R_{2}^{2}\ln(R_{2}/R_{1})}{R_{2}^{2}-R_{1}^{2}}\tau_{H}+\frac{4\pi L_{e}R_{1}^{2}R_{2}^{2}}{R_{2}^{2}-R_{1}^{2}}\eta$$

When there is no external magnetic field, the magnetorheological fluid exhibits the characteristics of a Newtonian fluid. $L_{1}$ is the circumferential length of the place; without the action of magnetic flux, viscous flow occurs, and its viscous resistance moment is

(6) $$T_{2}=\frac{4\pi L_{1}{R_{1}}^{2}{R_{2}}^{2}\omega }{{R_{2}}^{2}-{R_{1}}^{2}}\eta$$

In which $L_{1}=2\pi R_{1}$, the total torque can be obtained by formula (5) and formula (6)

(7) $$\begin{array}{l} T=T_{1}+T_{2}\\ =\frac{4\pi L_{e}{R_{1}}^{2}{R_{2}}^{2}\ln(R_{2}/R_{1})}{{R_{2}}^{2}-{R_{1}}^{2}}\tau_{H}+\frac{4\pi (L_{1}+L_{2}){R_{1}}^{2}{R_{2}}^{2}\omega }{{R_{2}}^{2}-{R_{1}}^{2}}\eta \end{array}$$

In the domain of haptic interface design, the concept of “Z-width” serves as a critical metric for evaluating system performance, specifically denoting the achievable impedance dynamic range of the haptic interface [29]. For haptic interfaces utilizing magnetorheological (MR) technology, this dynamic range is contingent upon the correlation between the input current and the system’s force output. The system’s maximum force output is dictated by the maximum shear stress of the MR fluid, which is influenced by both the maximum input current and the structural configuration of the actuator.

The maximum input current is constrained by several factors. Firstly, the saturation of the magnetic field strength plays a critical role. As the current reaches a certain threshold, the magnetic field ceases to increase significantly, thereby limiting the further alignment of the magnetorheological (MR) fluid particle chain structure and consequently affecting the force output. Additionally, the MR fluid itself exhibits chain structure saturation. Once the particle chain attains its limit, the force response cannot be further enhanced. Lastly, the high-temperature resistance of the coil cladding restricts the maximum permissible current, as excessive temperatures may lead to material failure or system damage.

Consequently, the dynamic range of the MR haptic interface is influenced by various constraints, including current input, material properties, and system design. This necessitates comprehensive trade-offs in the design process to optimize its performance.

## 5. Performance Evaluation

To assess the feasibility of the project, this section will design and conduct a performance evaluation experiment for the master haptic interface. Part (1) will evaluate the force feedback capability of the master haptic interface, Part (2) will assess its response performance, and Parts (3) and (4) will examine its haptic feedback performance. The magnetorheological fluid used in the master haptic interface was the commercial product MRF-A181 (Shenzhen POCE Technology Co., Ltd., Shenzhen, China).

### 5.1. Haptic Force Calibration and Hysteresis Characteristics Test

**Figure 6:**
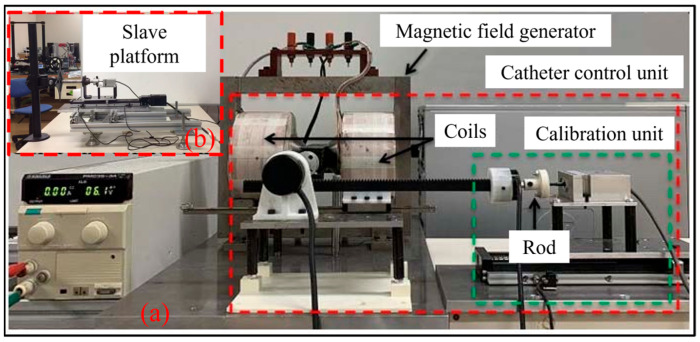
Experimental setup for haptic force assessment: (**a**) the proposed master haptic interface, (**b**) the slave platform.

To investigate the correlation between haptic force and input current, varying coil currents were administered to each coil, with the current range established from 0 to 3.8 A and a step increment of 0.20 A. The experimental configuration is depicted in Figure 6. During the experiment, it is crucial to maintain force feedback control between the haptic interface and the slave manipulator to precisely regulate the haptic force, ensuring it aligns with the reference force measured by the slave manipulator. Furthermore, a specialized experiment was conducted to validate the hysteresis characteristics of the catheter haptic interface. The total catheter insertion displacement of the experimental apparatus was set to 120 mm, with an insertion speed of 5 mm/s [28]. During the experiment, the input current was incrementally increased from 0 A to 3.0 A in steps of 0.20 A and subsequently decreased back to 0 A.

**Figure 7:**
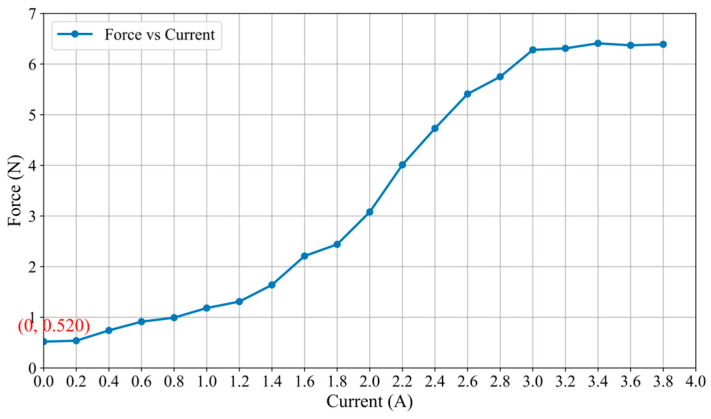
The haptic force and the input current.

**Figure 8:**
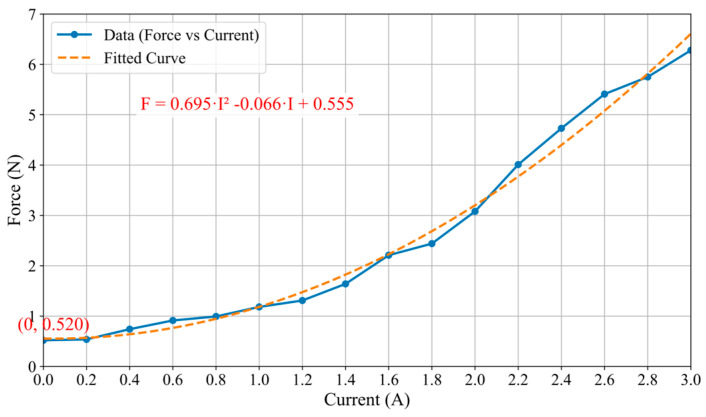
Relationship between the haptic force and the input current.

The relationship between the applied current and the resulting force, as illustrated in Figure 7, demonstrates a strong positive correlation. The force increases with the current over a significant portion of the measured range, from 0 A to approximately 3.0 A. The force value generated by the current exceeding about 3.0 A is basically maintained at about 6.28 N, indicating that the saturation effect may occur in the system. This clear functional dependency confirms the predictable and controllable nature of the force generation mechanism with respect to the input current. As shown in Figure 7 (Force Output vs. Current), the measured system’s inherent friction is a stable and repeatable value of 0.52 N (the output force at 0 A input current). This represents the combined, fixed resistance from the cylinder-MR fluid interface (in its zero-field state), the rack and pinion, and the linear slide. This stands in direct and favorable contrast to the force baseline in our previous prototype design [30]. In our previous prototype, the frictional force of the entire system was an unpredictable variable. This significant change is mainly caused by the uncertain and inconsistent friction between the operating rod and the orifice periphery in the MR fluid container wall, which is the main source of “external friction interference”. Our new design successfully eliminates this interference by redesigning the mechanical layout of the actuator, effectively minimizing variable external friction and generating purer and more reliable force feedback signals. Concurrently, a relationship curve between the haptic force and the input current was constructed based on the experimental data, as illustrated in Figure 8. The approximated curve can be obtained and can be expressed as follows:

(8) $$F=0.695I^{2}-0.066I+0.555$$

where F and I are the displayed force (called haptic force) and the input coil current, respectively. This relationship elucidates the substantial impact of input current on haptic force, laying the foundation for precise output of tactile force at the master haptic interface.

**Figure 9:**
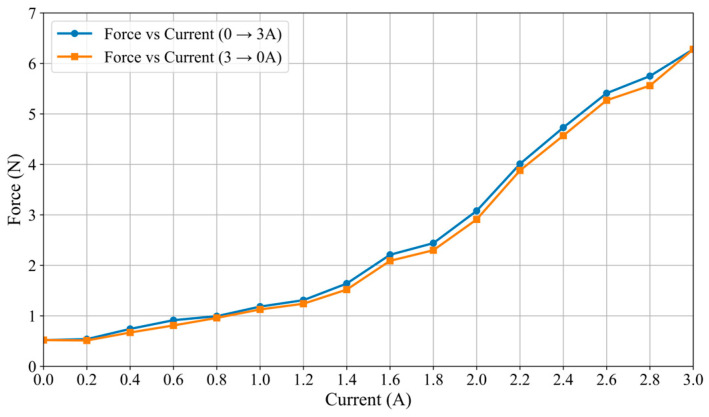
Hysteretic characteristics.

In the hysteresis characteristic test (see Figure 9), the catheter haptic interface demonstrates pronounced hysteresis. As the current increases from 0 to 3 A and subsequently decreases to 0 A, the haptic force varies along distinct paths in response to the current. This hysteresis phenomenon is primarily attributed to the hysteresis effect of the MR fluid and the system’s dynamic friction. The particle chain of the MR fluid is progressively established as the magnetic field intensifies; however, the unchaining process is gradual when the magnetic field diminishes, resulting in the haptic force lagging behind the current change during the reduction phase. Concurrently, the irreversibility of the dynamic friction force further exacerbates this hysteresis characteristic. Experimental data indicate that the haptic force increases more rapidly with rising current but exhibits a slower decline as the current decreases, thereby forming a distinct hysteresis loop.

The experimental results presented in this section are of great significance. Firstly, by formulating a nonlinear model correlating haptic force with input current, it is possible to achieve dynamic compensation of force output, thereby mitigating the effects of dynamic friction and hysteresis. Secondly, the hysteresis characteristics identified through the experiment offer valuable insights for the optimal design of catheter haptic interfaces. Enhancing the hysteresis performance of magnetorheological (MR) fluid or refining the electromagnetic control algorithm can effectively diminish the hysteresis phenomenon, thereby enhancing the response speed and control accuracy of haptic force feedback. These investigations provide theoretical support and a design framework for the practical application of catheter haptic interfaces, which is crucial for enhancing the precision of catheter control and ensuring surgical safety.

To further assess the performance limits of the proposed master haptic interface, we conducted a quantitative analysis of the system’s Z-width under varying input currents. In haptic systems, Z-width denotes the dynamic range of achievable impedance, specifically the system’s capacity to transition between high- and low-impedance states with precision and responsiveness. This parameter is essential for accurately simulating both free motion and constrained contact in surgical applications.

**Figure 10:**
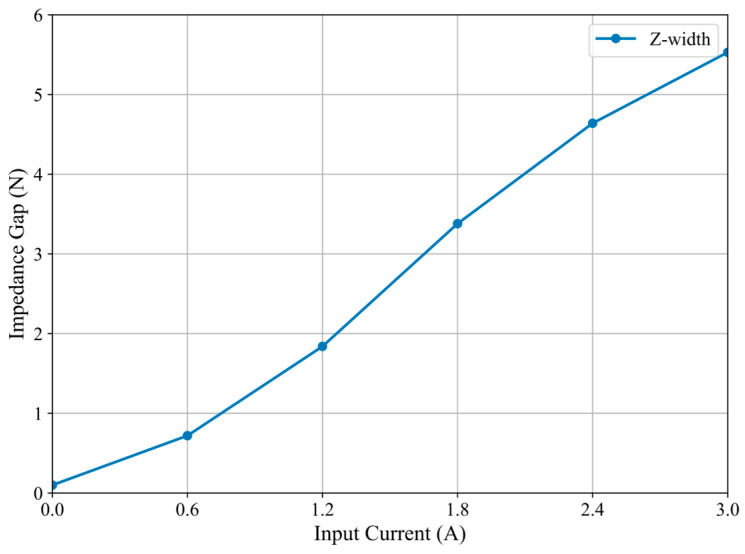
Z-width response of the haptic interface under increasing current.

To assess the Z-width, we measured the output force of the haptic interface under a series of predefined input currents ranging from 0 A to 3 A, with a step size of 0.2 A, under both static and dynamic conditions. The experiment was conducted without any external load to represent the “free-space” condition and with a constrained rigid surface to simulate the “contact” condition. The minimum impedance was recorded at 0 A, corresponding to the MR fluid in its Newtonian state. Conversely, the maximum impedance was observed at 3 A, indicating the MR fluid was in a solid-like state. The force differential under these conditions, at identical displacement, was employed to approximate the Z-width. As illustrated in Figure 10, the Z-width of the haptic interface exhibits a nonlinear increase with the input current. At an input of 3A, the impedance gap attained a value of 5.53 N, signifying a broad range of force modulation. This finding substantiates that the proposed system possesses a high-fidelity impedance bandwidth, enabling it to effectively simulate scenarios involving both soft tissue insertion and hard collision.

To elucidate the formation mechanism of the previously mentioned Z-width curve, this section will build upon the magnetorheological fluid (MRF) shear model developed in Section 4. Beginning with the stress–strain relationship and employing the torque superposition principle, it will elucidate the system’s impedance variations with and without the influence of a magnetic field and validate the model’s predictive accuracy through experimental results. In the mechanical model presented in Section 4, the stress–strain relationship of magnetorheological (MR) fluid is described by the Bingham model, as represented in formula (1). Under magnetic stimulation, the impedance increases significantly, resulting in a high-impedance state; in the absence of the field, the fluid behaves as a Newtonian fluid with minimal resistance. Based on this formulation, the system’s output torque under a magnetic field can be derived and incorporated into expressions (2) through (5), yielding the torque generated by MR fluid shear resistance. Conversely, the Newtonian resistance moment is characterized by Equation (6), corresponding to a low-impedance baseline. Consequently, the total torque output from the master haptic interface is a superposition of the MR-induced torque and the Newtonian viscous torque, as expressed in Equation (7). The concept of Z-width—defined as the achievable impedance range—is thus directly related to the system’s parameters. The theoretical maximum and minimum force outputs of the system can be derived as follows:

(9) $${Z-width}_{\tau }=\tau_{\mathrm{total}}^{\max}-\tau_{\mathrm{total}}^{\min}=\tau_{H}$$

(10) $${Z-width}_{M}=M_{\mathrm{total}}^{\max}-M_{\mathrm{vis}}=M_{\mathrm{mr}}$$

The relationships elucidate that yield stress is the primary determinant affecting Z-width. As the input current increases, the yield stress rises due to the intensification of magnetic fields, thereby augmenting the MR fluid’s resistance to motion. Consequently, the system exhibits an expanded dynamic impedance range. In the experimental validation presented in this section, the force output was measured under varying input currents, corresponding to different magnetic field strengths. The resultant Z-width curve, as shown in Figure 10, corroborates the theoretical predictions derived from the Bingham model. The strong correlation between theoretical torque modeling and empirical force measurements substantiates the efficacy of the haptic interface design in providing adaptive force feedback.

### 5.2. The Haptic Force Response Speed Test

To assess the efficacy of the master-side feedback haptic prompt in collision detection, a dynamic force evaluation experiment was devised and executed to quantitatively analyze the magnitude of the feedback haptic force and its impact on the operator during initial operation. The experimental setup is depicted in Figure 6a, where a rigid rod is stably driven by a slider, and force data is recorded using a load cell (TEAC, TU-UJ, Japan). The insertion motion of the rigid rod is generated within the haptic interface, and the force data, including the collision force at the endpoint and the haptic force relayed by the master, are collected and displayed in real-time by a PC system. To ensure the accuracy of the force feedback and minimize external interference, the insertion motion’s input speed is set at 5 mm/s, which guarantees the stability and repeatability of the measurement data without affecting the haptic force [28]. To maximize the haptic force, in conjunction with the findings of Experiment 1, a large current of 3.0A is employed in this experiment to generate the strongest magnetic field strength. Under the influence of this magnetic field, the MR fluid rapidly transitions from a liquid to a structured state with solid properties, thereby significantly enhancing the output of haptic force [31]. This design ensures a substantial haptic feedback force, providing optimal conditions for validating the effectiveness of collision detection.

**Figure 11:**
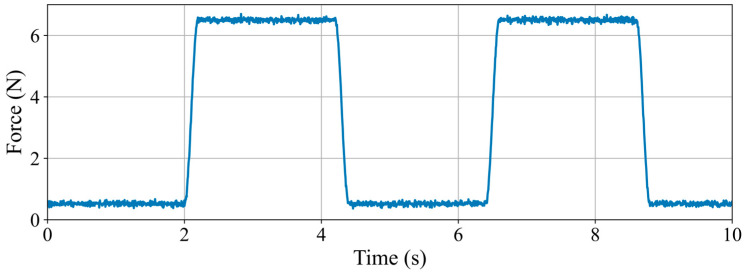
The experimental results for haptic force response speed.

The experimental results are depicted in Figure 11, which clearly illustrates the dynamic variation process of haptic force. Upon the application of the electromagnetic field, the dynamic force rapidly escalates from its initial value to the maximum value, indicating the system’s high sensitivity to external collision signals. In the experiment, it was measured that when the master haptic interface is powered on, the haptic feedback force will rapidly increase to approximately 6.28 N within 25 milliseconds, which significantly exceeds the haptic resolution of human fingers (minimum perceptible difference, JND), approximately 19 mN [32]. This result demonstrates that the change in haptic force is not only substantial but also capable of quickly triggering the operator’s stress response, thereby achieving effective collision detection.

Furthermore, the experiment corroborated that even novice operators can rapidly discern changes in haptic feedback force. This substantial alteration in haptic force allows operators to promptly implement measures to prevent further collisions or damage, thereby significantly enhancing the safety and fault tolerance of system operations. The haptic interface, which is based on magnetorheological technology, has demonstrated exceptional performance in collision detection and force feedback. This capability is beneficial not only for skilled operators but also proves effective for those with limited experience.

The experimental findings indicate that the rapid response and substantial variations in haptic force are crucial for achieving real-time collision detection. The integration of the rapid solidification properties of MR fluid with the maximum current input ensures the efficacy and stability of the haptic feedback force. These studies offer a robust theoretical foundation and empirical support for the future optimization of haptic feedback systems in the medical field (such as catheter manipulation in minimally invasive surgery) and the industrial sector (such as collision warning in complex mechanical operations). Future research may further investigate the behavior of haptic force under varying magnetic field strengths and input speeds to enhance the system’s adaptability and operational precision.

### 5.3. Feedback Force Test Under Manual Operation

**Figure 12:**
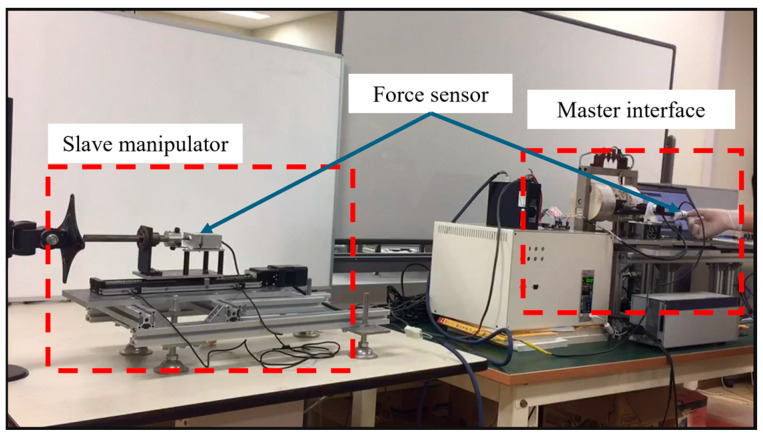
Experimental setup for the haptic feedback and the collision alert testing.

To evaluate the project’s feasibility, a feedback force test experiment was executed. This experiment involved two components: a feedback force test conducted under manual operation and a force stability test. For axial motion, a rotary encoder equipped with a pinion and a rack was employed as the input and feedback mechanism, as shown in Figure 12. Similarly, for radial motion, another rotary encoder was utilized as the input and feedback mechanism. The positions of these encoders at the master end were decoded and relayed to the motion controllers, ensuring that the slave robot accurately mirrored the axial and radial positions of the master end. The Arduino Due development board (Smart Projects, Strambino, Italy) incorporates a 32-bit ARM architecture microcontroller, facilitating the real-time orthogonal decoding and transmission of positional data to the control unit. Concurrently, the resistance on both the master and slave sides was quantified using a force sensor affixed to the drive shaft.

Figure 12 illustrates the experimental configuration for the feedback force assessment conducted under manual operation. During the experiment, the force exerted on the catheter was continuously measured by the catheter manipulator, while the feedback force was generated by the master haptic interface and conveyed to the operator. In this setup, the catheter was affixed to a slider and moved horizontally to replicate actual operating conditions. The operator controlled the catheter’s movement by manipulating the master haptic interface at the master end, and the slider precisely mirrored the operator’s actions at the slave end, thereby establishing a complete master–slave control closed loop. The master haptic interface provided immediate control information through force feedback, enabling the operator to perceive the force applied to the catheter.

To acquaint the operator with the experimental procedure and the proposed haptic feedback method, adaptive exercises were conducted before the formal test. Initially, the operator engaged in multiple manual operations, followed by robot operation training with the aid of feedback force. During the formal experiment, the operator utilized the feedback force assistance system to perform the same task as in the manual operation. The experimental protocol established an operating force threshold of 1.2 N. Upon surpassing this force value, the master haptic interface increased the feedback force to alert the operator. Upon detecting a collision warning, the operator will immediately cease advancing the operation rod. This action was reinforced by the master haptic interface, which generated a maximum resistive force to physically prevent further forward movement. The operator would then retract the operation rod to reduce the contact force, adjust the insertion angle, and reattempt the operation. This cycle was repeated multiple times to rigorously evaluate the consistency and effectiveness of the collision alert system.

**Figure 13:**
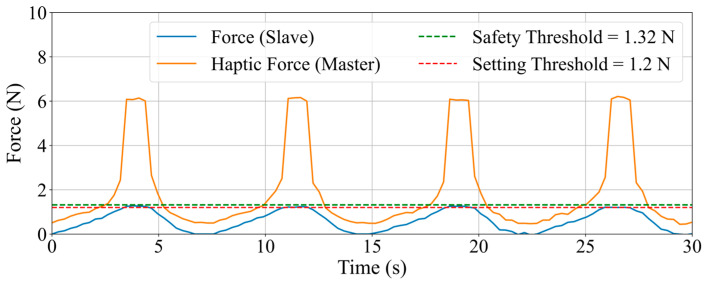
Experimental results of real-time force feedback and collision alert.

The experimental results are shown in Figure 13, which plots the forces from the slave side, the master-side haptic forces, along with the safety threshold and the setting threshold. In the figure, the green dashed line represents the safety threshold, which is set at 1.32 N in this experiment, while the red dashed line represents the set threshold, configured at 1.2 N for this study. When the contact force at the slave side is below the setting threshold, the haptic feedback force generated at the master side varies in accordance with changes in the slave-side contact force, maintaining a difference of approximately 0.52 N, which corresponds to the dynamic friction force of the master haptic interface in the absence of current. When the slave-side contact force exceeds the setting threshold, the controller promptly increases the master-side haptic feedback force to approximately 6.28 N, providing the operator with a clear haptic collision warning signal. When the operator performs a retraction maneuver, causing the slave-side contact force to fall below the setting threshold, the master-side feedback force rapidly decreases and provides real-time feedback at a value approximately 0.52 N greater than the slave-side contact force. In this experiment, when the operator received a high-resistance haptic reminder to stop the operation and advancement, the contact force from the slave side was within the safety threshold. This timely and pronounced haptic feedback mechanism effectively alerts the operator to promptly modify the operational strategy or halt catheter advancement, thereby significantly reducing the risk of vascular tissue damage and enhancing the safety and controllability of vascular interventional procedures.

### 5.4. In Vitro Evaluation

**Figure 14:**
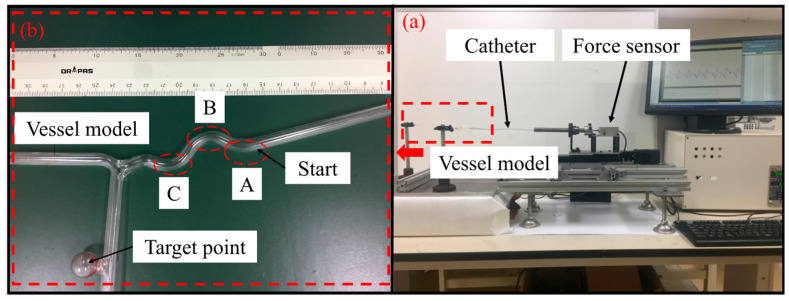
Experimental setup for in vitro test: (**a**) the slave platform, (**b**) the blood vessel model.

To further validate the efficacy of the master haptic interface designed in this study for enhancing the safety of interventional surgical procedures, an in vitro intubation test was conducted. The experimental setup is depicted in Figure 14a. In this experiment, volunteers were tasked with operating the master haptic interface, while the slave robot platform maneuvered the catheter to sequentially reach predetermined target points within the vascular model, as illustrated in Figure 14b. As shown in Figure 14b, the vascular model path comprises a straight segment and several small curved segments, with a total motion length of approximately 15 cm. The experiment was divided into two groups: (1) a control group, where volunteers completed the catheterization task relying solely on visual feedback without haptic feedback; and (2) an experimental group, where volunteers performed the same task with both visual and haptic feedback. The master haptic interface continuously senses the force applied to the catheter. As the contact force on the slave side increases, the feedback force on the master side also increases. When the contact force on the slave side exceeds the set threshold, the haptic feedback force on the master side rapidly increases to the maximum value (approximately 6.28 N) to alert the operator of a catheter tip collision. The intubation angle of the catheter should be adjusted promptly to maintain the catheter contact force within the safety threshold, thereby reducing the contact force between the catheter and the vascular wall during intubation and mitigating the risk of vascular injury.

In this experiment, ten volunteers (non-medical) were recruited to perform catheter insertion tasks under two distinct operating conditions. Each participant was required to maintain the catheter contact force within a specified safety threshold range. The experimental conditions were as follows: a threshold of 1.2 N and a safety threshold of 1.32 N were established at the first bend, A, of the vascular model. As the insertion depth and number of bends increased, the threshold increased by 0.15 N at each subsequent bend position due to the rise in frictional resistance. Specifically, the threshold set at bend B was 1.35 N with a safety threshold of 1.47 N, and at bend C, the threshold was 1.5 N with a safety threshold of 1.62 N. To ensure the reliability and repeatability of the experimental data, each operation was repeated ten times under each condition. The following two important metrics were considered in this experiment: (1) the average elapsed time of the task accomplishment and (2) the frequency of exceeding the safety threshold. These key data points were utilized to compare the operational efficiency and safety of the two task groups and to comprehensively evaluate the auxiliary role of the master haptic interface in catheterization tasks.

**Figure 15:**
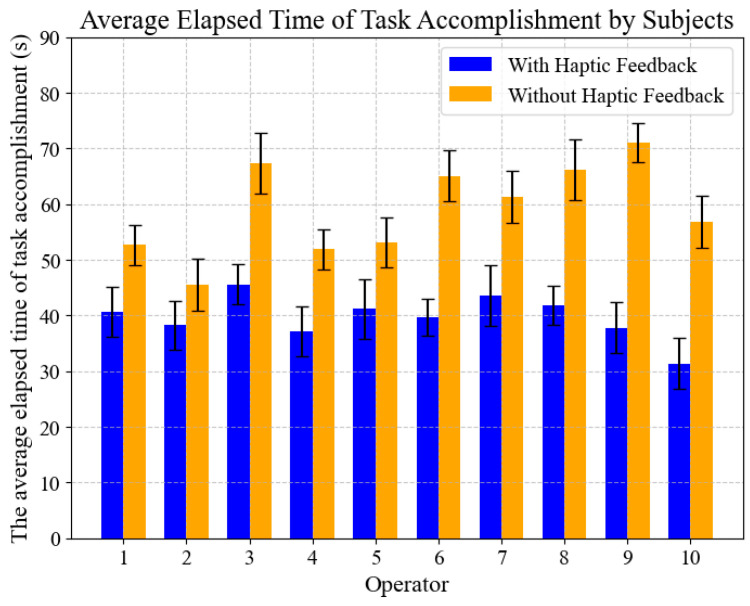
The average elapsed time of task accomplishment.

Figure 15 shows the average elapsed time of task accomplishment for all participating operators. The statistical results indicate that the average elapsed time of the experimental group is shorter than that of the control group. The primary rationale for this reduction is that haptic feedback can diminish the time required to monitor contact force and alleviate the psychological pressure on the operator caused by delayed collision responses. The system enables the surgeon to manipulate the catheter with greater attentiveness and fluidity, thereby decreasing the duration of the procedure. Consequently, the reduction in operating time can lessen the duration of radiation exposure for the patient, thereby mitigating radiation-induced harm to the human body.

**Figure 16:**
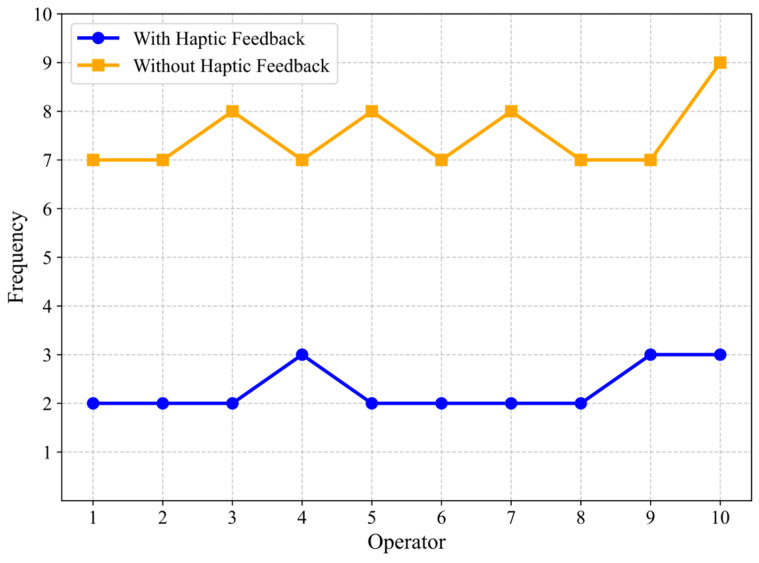
The frequency of exceeding the safety threshold.

Further analysis of the results presented in Figure 16 reveals that the frequency with which the catheter tip contact force applied by the operator in the experimental group exceeded the safety threshold was significantly lower than that observed in the control group. This can be attributed to the quick reaction required to adjust the catheter’s position following a tip collision. During this brief interval, even a slight forward displacement of the catheter can exacerbate the collision. This scenario is highly dependent on the technical proficiency and psychological resilience of the operators. However, when a collision is communicated to the operator through haptic feedback, the response time is minimized. Furthermore, the master haptic interface in this system not only alerts the operator to collisions but also generates a sudden viscous resistance at the master-side operation rod, preventing further forward movement of the catheter. This mechanism reduces the continued increase in collision force and enhances the operational safety margin. Nevertheless, it was observed that contact forces could still occasionally exceed the predefined safety threshold, even with the haptic collision alerts activated, though such occurrences were significantly less frequent compared to scenarios without haptic feedback. This phenomenon can be attributed to two primary factors. First, the operators involved in the experiment were individuals without medical backgrounds or prior training. As a result, there were noticeable variations in their perceptual sensitivity to haptic cues and their corresponding reaction speeds. Second, mechanical imperfections, specifically the presence of meshing backlash in the rack-and-pinion mechanism, could occasionally lead to sudden jamming or uneven motion transmission during operation. Such unexpected resistance changes could delay the operator’s response, resulting in a momentary overshoot of the contact force. Regarding the first issue, subsequent improvements in response consistency are anticipated with increased operator familiarity and repeated training. To address the second issue, future iterations of the device will incorporate a dual-gear anti-backlash mechanism, designed to eliminate transmission play and ensure smoother, more predictable operation.

The proposed master haptic interface with collision alert functionality has the potential to significantly reduce operating time and promptly notify the operator of any collisions. This capability enables the operator to swiftly adjust the catheter, thereby mitigating the risk of vascular perforation due to collisions and enhancing the overall safety of the procedure.

## 6. Conclusions

This paper introduces a novel master haptic interface engineered for the precise transmission of motion information during catheter/guidewire manipulation in minimally invasive surgical procedures. Utilizing magnetorheological (MR) fluid and high-precision force sensors, the interface provides real-time haptic cues and collision warnings, significantly enhancing surgical safety and operational precision. Its key advantage is to minimize the external friction interference of the magnetorheological fluid container, delivering pure and reliable force feedback signals. Experimental validation demonstrated that the dynamic haptic forces generated by the interface exhibit significant and clearly perceptible changes, sufficient to trigger operator response. Crucially, near the preset force threshold, the interface effectively amplifies the feedback force to provide an early warning, substantially reducing the risk of threshold exceedance. Furthermore, in in vitro experiments, the introduction of haptic feedback significantly reduced the operator’s average completion time, yielded smaller and more stable applied forces, and markedly decreased the frequency of threshold exceedance. This not only minimizes potential damage to simulated vasculature but also substantially improves procedural smoothness and safety. In summary, by integrating force feedback, haptic cues, and collision warning functionalities, this master haptic interface effectively leverages the operator’s innate skills while compensating for the absence of haptic information in conventional systems. It provides crucial technical support and informs optimization strategies for the design of future intravascular robotic systems, opening new avenues for the development and application of minimally invasive surgery.

In the future, we will complete a specialized slave manipulator design, following which we will conduct comprehensive in vitro simulated surgical procedures. These future experiments are specifically planned to evaluate the entire system’s performance, including the realism of the haptic feedback, under conditions that closely mimic actual clinical tasks.

## Data Availability

Data are contained within the article.
